# Advancements in Spinal Endoscopic Surgery: Comprehensive Techniques and Pathologies Addressed by Full Endoscopy Beyond Lumbar Disc Herniation

**DOI:** 10.3390/jcm14113685

**Published:** 2025-05-24

**Authors:** Jad El Choueiri, Francesca Pellicanò, Edoardo Caimi, Francesco Laurelli, Leonardo Di Cosmo, Ali Darwiche Rada, Daniel Cernigoi, Arosh S. Perera Molligoda Arachchige, Giorgio Cracchiolo, Donato Creatura, Ali Baram, Carlo Brembilla, Gabriele Capo

**Affiliations:** 1 Humanitas University, Via Rita Levi Montalcini 4, 20072 Pieve Emanuele, Milan, Italy; francesca.pellicano@st.hunimed.eu (F.P.); edoardo.caimi@st.hunimed.eu (E.C.); francesco.laurelli@st.hunimed.eu (F.L.); leonardo.dicosmo@st.hunimed.eu (L.D.C.); ali.darwiche@st.hunimed.eu (A.D.R.); daniel.cernigoi@st.hunimed.eu (D.C.); aroshperera@outlook.it (A.S.P.M.A.); 2 School of Medicine and Surgery, University of Milano-Bicocca, 24127 Bergamo, Milan, Italy; g.cracchiolo@campus.unimib.it; 3 Department of Neurosurgery, IRCCS Humanitas Research Hospital, Via Manzoni 56, 20089 Rozzano, Milan, Italy; donato.creatura@humanitas.it (D.C.); ali.baram@humanitas.it (A.B.); carlo.brembilla@humanitas.it (C.B.); gabriele.capo@humanitas.it (G.C.); 4 Department of Biomedical Sciences, Humanitas University, Via Rita Levi Montalcini 4, 20072 Pieve Emanuele, Milan, Italy

**Keywords:** endoscopic spine surgery, minimally invasive, spine management, UBE—unilateral biportal endoscopy, FESS—full-endoscopic spine surgery

## Abstract

Endoscopic spine surgery (ESS) has traditionally been employed for lumbar disc herniation (LDH). Recent innovations in surgical methods and technologies have expanded its range to address other spinal pathologies, providing minimally invasive solutions with potential clinical benefits. Our review aims to summarize the applications, clinical outcomes, and limitations of ESS beyond LDH, focusing on its role in complex spinal conditions such as stenosis, thoracic disc herniation, spinal tumors, synovial cysts, and failed back surgery syndrome. A thorough review of the literature was conducted to assess and summarize the current evidence regarding ESS applications for spinal conditions beyond LDH surgery. Areas of focus included innovations in technology and technique, as well as comparisons with conventional open surgical methods. ESS shows notable potential across different spinal conditions by providing minimally invasive alternatives to traditional open surgery. Its use could be associated with reduced surgical morbidity, shorter recovery times, and improved patient outcomes. In particular, ESS is versatile in addressing both degenerative and neoplastic conditions of the spine. Despite this, challenges such as technical complexity, steep learning curves, and limited indications for certain pathologies remain as barriers to wider adoption. ESS is evolving in spine surgery, extending its utility beyond LDH surgery. While the current evidence largely supports its clinical efficacy, further studies are needed to address the present limitations and optimize its application. Future developments in surgical training and technology will likely enhance its adoption and broaden its clinical indications.

## 1. Introduction

Endoscopic spine surgery (ESS) is revolutionizing the surgical standards of various traditional procedures, providing a minimally invasive alternative to open surgery. Its appeal lies in its ability to achieve clinical outcomes comparable to those of open surgery while offering additional benefits such as minimized soft tissue disruption [1], shorter hospital stays [2], and fewer postoperative infections [2,3]. These advantages are very valuable, particularly in our aging population, where a less invasive approach can mitigate surgical risks associated with comorbidities and enhance recovery time. The development of advanced endoscopic systems has facilitated more precise interventions, allowing surgeons to address pathologies that were previously challenging to approach using endoscopic techniques [4].

Despite the significant advancements in ESS, its early development was largely limited to addressing lumbar disc herniation. Its origins can be traced back to the late 20th century, pushed by the pioneering work of Parviz Kambin who introduced “Kambin’s triangle” [5], a safe working zone in the spine for accessing herniated discs, and this was notable in the evolution of ESS [6]. Early procedures, such as endoscopic lumbar discectomies, were primarily focused on removing herniated disc material in patients with radicular pain, offering a less invasive alternative to laminectomies. In parallel, growing interest in discogenic pain mechanisms and non-compressive disc pathology contributed to the refinement of surgical indications for lumbar ESS [7].

**Figure 1:**
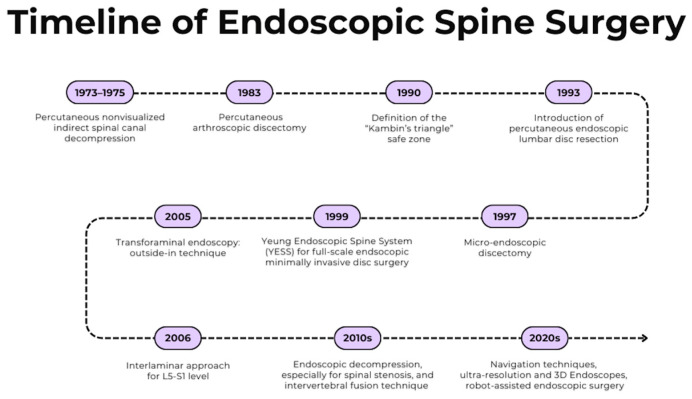
Diagram summarizing the advancements in the field of endoscopic spine surgery over time (created by the authors with Canva.com).

As surgeons became more experienced and as this technology evolved with better optics, high-definition cameras, and specialized instruments, the potential for ESS to treat more complex pathologies became apparent over time (Figure 1). ESS currently incorporates new technologies, such as navigation [6], augmented and virtual reality, robotics [8], and three-dimensional and high-resolution imaging of neuro-anatomical structures [9], leading to improved outcomes [10]. As a result, its applications have expanded to a wide array of complex spinal conditions, including spinal stenosis, spondylolisthesis, spinal deformities, tumors, infections, synovial cysts, and other degenerative conditions [11].

Indeed, surgeons now have the ability to perform precise procedures with minimal damage to surrounding tissues, which is particularly beneficial in complex cases where invasive surgeries were traditionally required [12]. It remains important to note that endoscopic spine surgery is part of a broader group of minimally invasive spine surgery techniques [1]. These include percutaneous pedicle screw placement, which involves placing screws through small skin incisions under radiological guidance; minimally invasive transforaminal interbody fusion; minimally invasive posterior cervical foraminotomy, which is used to decompress nerve roots through small incisions via specialized instruments; robotic spine surgery; and many other procedures [13].

This literature review aims to discuss the current applications of ESS in surgically treated pathologies beyond lumbar disc herniation and provides a comprehensive overview of its expanding indications, clinical outcomes, and future directions.

## 2. Evolution of Endoscopic Instrumentation

Techniques and instrumentation in spinal endoscopic surgery have experienced significant advancements over the years, particularly with the shift from traditional endoscopic methods to full-endoscopic approaches. Initially, spinal endoscopic surgery was performed without the irrigation of saline solution, a practice that often limited the visibility within the surgical field [14]. The introduction of irrigation marked a critical turning point, as continuous saline flow not only improved visualization by clearing blood and tissue debris but also provided a safer and more controlled environment, minimizing thermal damage from the endoscopic instruments [15]. This innovation paved the way for what is now referred to as “full-endoscopic” surgery (FESS), distinguished from the earlier “endoscopic” techniques by its enhanced imaging clarity and access to deeper spinal structures [16].

A further development in spinal endoscopic surgery is the distinction between uniportal and biportal (UBE) instrumentation. Uniportal endoscopy uses a single entry point, allowing for a streamlined approach that reduces the amount of tissue disruption and has a shorter learning curve for surgeons already familiar with minimally invasive techniques [17]. On the other hand, biportal endoscopy utilizes two separate entry points—one for the endoscope and the other for the working instruments. This setup allows for greater maneuverability within the surgical field and enables more complex procedures, as instruments can operate independently of the endoscope [18]. However, the biportal approach may require a higher level of expertise and is often associated with slightly increased soft tissue manipulation [19].

These technological advances have not only expanded the range of treatable spinal pathologies, but also brought about significant benefits for patient outcomes, such as reduced postoperative pain, faster recovery, and minimized risk of infection [20]. The continuous evolution of both uniportal and biportal techniques, along with improvements in endoscopic visualization and irrigation systems, underscores the dynamic and advancing nature of spinal endoscopic surgery, setting a robust foundation for its application across a broader spectrum of spinal conditions, explored in the following sections.

## 3. Spinal Stenosis

Spinal stenosis is a degenerative condition characterized by the narrowing of the spaces around the spinal neurovascular structures due to changes in ligamentum flavum, facet joints, and eventually, the spinal disc [21]. Lumbar spinal stenosis is one of the most common reasons for spinal surgery in the elderly population, significantly affecting quality of life due to chronic pain, disability, and impaired mobility [22]. The hallmark clinical sign of LSS is neurogenic claudication [23], which is described as leg pain during walking that is relieved by lumbar flexion or sitting. 

Non-surgical treatments, such as physiotherapy, medication, spinal injections, and lifestyle modifications, are typically the first line of treatment [24], but many patients fail to achieve long-term relief [25]. Notably, there seems to be limited and contradictory evidence regarding whether patients with moderate pain benefit more from surgery or from conservative treatment [26]. When conservative therapies prove ineffective, surgery becomes an option, with the primary goal being the decompression of the central spinal canal and neural foramina to relieve pressure on the spinal nerves.

Traditional surgical decompression, such as partial laminectomy, with or without fusion, has long been the standard treatment for lumbar spinal stenosis (LSS) [27]. The benefit of lumbar decompression has been confirmed in several studies [28]. However, these procedures may be associated with significant drawbacks [29], including a lengthy recovery, potential for spinal instability, and the need for reoperations. Additionally, the inclusion of spinal fusion to prevent instability remains controversial, particularly in patients without accompanying spondylolisthesis [30]. As a result, modern minimally invasive techniques, including endoscopic decompression surgery, have gained traction as less invasive and more effective alternatives [31].

Endoscopic spine surgery is thus an alternative with potential advantages over open procedures. The technology allows direct visualization of pain generators, enabling surgeons to precisely target the areas that require decompression [32]. Transforaminal endoscopic decompression, for instance, has been reported to be effective in treating foraminal stenosis, particularly in patients who are poor candidates for general anesthesia or those at higher surgical risk due to age or comorbidities [33]. The adverse events do not differ statistically with those associated with an open approach [34], but there is evidence of better muscle preservation and better pain relief [35]. Endoscopic surgery can help spare the ligaments and the articular process, which may prevent the need for reoperation due to postoperative instability [36] when compared with the results of the traditional approach.

Patient selection is critical in determining the success of endoscopic approaches [37]. Nevertheless, recent advances in endoscopic technology have expanded the potential applications of these techniques in treating LSS. Overall, while traditional open decompression surgery, such as laminectomy, remains a reliable and well-established treatment, modern minimally invasive and endoscopic techniques are increasingly recognized for their ability to achieve similar clinical outcomes with fewer complications.

## 4. Thoracic Disc Herniation

Symptomatic thoracic disc herniation (TDH) is a condition where, in the thoracic segment of spinal column, the nucleus pulposus of an intervertebral disc slides through a breach in its corresponding annulus fibrosus and protrudes into the spinal canal, putting pressure on that level of the spinal cord. TDH is a quite rare condition, many times less likely than lumbar disc herniation (LDH) [38]. This low rate of incidence is due to the unique properties of the thoracic segment: as the vertebrae are connected to the ribcage, the former supports and stabilizes the latter, also making it the least mobile segment of the spinal column. This puts its intervertebral discs under significantly less stress than the other segments. Moreover, most TDH cases are usually treated without the need for surgical interventions [38,39].

However, it is important to emphasize that the nature of TDH also makes it a more serious lesion, compared to LDH, if surgery is required. TDH surgeries are associated with high risk of morbidity [40]. Because the lesion is in such close proximity to the spinal cord, lungs, and important vessels, TDH surgeries are very technically demanding and may give rise to serious complications, such as dural tear, vascular or pulmonary damage, and subarachnoid-pleural fistula [41,42]. Moreover, TDH is characterized by a distinctly high frequency of disc calcification (42%) [43], which tends to add another layer of difficulty to the surgery [44].

Concerning endoscopic interventions for TDH, three techniques stand out.

The most popular approach is transforaminal endoscopic thoracic discectomy (TETD), as it can require only local anesthesia and results in the lowest rate of morbidity [45]. The surgery consists of accessing the herniated disc through the intervertebral foramen and is performed in the following three steps: first, discography, to properly identify the structures being assessed, then foraminoplasty, widening the intervertebral foramen until the extruded disc is exposed, and then disc removal, resulting in immediate decompression of the spinal cord. This procedure is performed with the use of a discographic needle, manual reamers or drills, and forceps [42].

TETD is very similar to its lumbar counterpart; however, peculiarities of the thoracic spine make it a more complex and difficult surgery. These include less free space in the spinal canal for the spinal cord, lower amounts of CSF, and smaller intervertebral foramina (hence, the need for foraminoplasty). In any case, TETD has shown to yield as good as, if not better, results than the traditional open thoracic discectomy.

An alternative approach is the endoscopic interlaminar technique [46]. Similar to TETD, it consists of inserting the probe into the interlaminar foramen; widening it by dissecting the surrounding laminae, ligamentum flavum, and vertebral joint until the spinal canal can be entered; and the herniated disc is then accessed and removed. The interlaminar technique is applied the same way in the thoracic and lumbar segment [47].

The transthoracic retropleural approach is mostly considered for larger, more medial or calcified disc herniations [46]. It is a unique technique, where the endoscopic instruments are inserted into an intercostal muscle at the posterolateral side and travel through the retropleural space until the disk is reached from the pedicle’s side. The head of the rib, pedicle, and epidural space need to be resected in this procedure. This method has no analog for LDH endoscopic surgery [47], and despite it being a more complex and unorthodox approach, it has proven to be a reliable and a minimally invasive technique [47].

Although it displays a significant learning curve and is very technically demanding, ESS seems to be a very effective intervention method for symptomatic thoracic disk herniation, with a wide range of effective approaches [47]. These satisfactory outcomes are attributed to the minimally invasive nature of the surgical techniques [48]. TETD, for example, often requires only local sedation and anesthesia, leaves thoracic structures well preserved, and results in minimal postoperative pain [42]. This is reflected in a meta-analysis by Sofoluke et al. [48], who found a diminished rate of complications and the need for prolonged hospitalizations. The authors pointed out that full endoscopic spine surgery has the capacity to change the standard of care of TDH treatment. 

It is important to stress that ESS use for symptomatic TDH is still a considerably unexplored field. With symptomatic TDH itself being a rare condition [38], it is only natural that studies on the topic will not be common. This translates into the slow development, refinement, and dissemination of new surgical techniques for its treatment. Nevertheless, even with a relatively small body of work, the few studies that discuss the use of ESS to treat TDH seem to overwhelmingly agree on its potential to provide better clinical outcomes in contrast to that of open surgery.

## 5. Spondylolisthesis and Degenerative Conditions

Spondylolisthesis refers to slippage of one vertebra with respect to another and is characterized by compressive radicular pain and neurological dysfunction [49]. The symptoms of the condition can present from mild to severe, depending on the degree of vertebral displacement and its respective influence on the adjacent structures. Any pathological process mediating the ability of the vertebral column to remain aligned may predispose to spondylolisthesis, and thus, with an aging population, the prevalence of the pathology is on the rise [50]. 

Treatment options primarily revolve around the severity of the condition, with low-grade cases often managed conservatively through physical therapy, steroidal pain management, and lifestyle modifications [51]. However, as the severity of the displacement increases or symptoms worsen irreversibly, more invasive interventions become necessary to alleviate symptoms [52].

In cases of moderate to high-grade spondylolisthesis, surgical intervention becomes the primary tool to stabilize the vertebral column and relieve nerve compression [53]. The principal surgical approaches are those involving decompression and fusion. Decompressive procedures such as laminectomy, laminotomy, or foraminotomy function by removing the bone or tissue impinging spinal nerves in an attempt to alleviate compression, while fusion attempts to stabilize the affected vertebrae by fusing them together, often using metal implants, bone grafts, ceramics, or other materials such as PEEK or BMPs. These procedures can occur in concomitance, or in some cases, decompression alone is preferred. 

Common fusion techniques like posterior lumbar interbody fusion (PLIF), transforaminal lumbar interbody fusion (TLIF), and anterior lumbar interbody fusion (ALIF) are effective for pain relief and function improvement [54,55] but are more invasive standard approaches, with minimal soft tissue preservation. They carry risks such as hematoma, infection, and adjacent segment disease [52], and this has led to interest in less invasive alternatives. These procedures are especially beneficial for severe neurological deficits and neuropathic pain but often involve prolonged recovery times.

**Figure 2:**
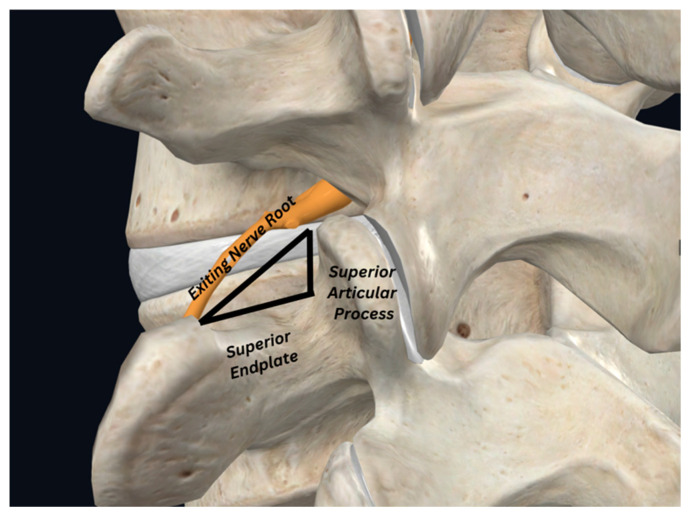
Kambin’s triangle (modified by the authors from *Complete Anatomy*, under a student license).

Endoscopic spine surgery has thus gained traction as a less invasive option for treating spondylolisthesis, offering several advantages over traditional methods [56]. As seen across the neurosurgical field, endoscopic techniques reduce the surgical footprint, keeping tissue disruption to a minimum, and resulting in decreased reported postoperative pain, quicker recovery, and fewer complications in the healing process [55]. The research of Soo Youn et al. and other comparative papers reveal that patients undergoing endoscopic decompression for spondylolisthesis experience faster returns to daily activities, shorter hospital stays, and reduced surgical times compared to those receiving open surgery [57]. Furthermore, the recently developed endoscopic techniques show promising surgical outcomes in stabilizing the spine; for example, the trans-Kambin’s triangle (Figure 2) lumbar interbody fusion has demonstrated similar patient outcomes to those for traditional techniques, offering a valuable minimally invasive alternative for both decompression and fusion [58,59,60]. Through the endoscopic approach, the ability to perform these procedures under local anesthesia further enhances patient comfort and reduces anesthesia-related risks [61]. 

Despite these advances, the integration of endoscopic spinal surgery as the gold standard for spondylolisthesis faces several obstacles. One of the primary difficulties is the steep learning curve for surgeons, as both the techniques and the equipment require extensive training and experience to gain proficiency with their use. Moreover, while endoscopic procedures are highly effective in some cases of spondylolisthesis, their application in more severe cases, requiring navigating more complex deformities, remains unclear, with traditional open surgery continuing to offer better surgical visibility and anatomical navigation to deal with problems arising from the anatomical variations present in the aforementioned cases [62,63]. Regardless, long-term data comparing endoscopic techniques to traditional methods are still needed to establish the long-term clinical outcomes of these procedures [56]. With further research and innovation, endoscopic spinal surgery has the potential to become a standard option for a broader range of clinical presentations within spondylolisthesis, encompassing higher grade deformities and vertebral displacements.

Nevertheless, ongoing technological advancements, such as the integration of 3D navigation systems, robotic assistance, and augmented reality [64,65], hold promise for improving the precision, safety, and applicability of endoscopic surgeries [66].

Among other degenerative conditions, synovial cysts have also been effectively treated with endoscopic surgery. Synovial cysts are fluid-filled sacs that arise from the synovial lining of facet joints in the spine, often due to degenerative changes [67]. These cysts can cause symptoms such as radiculopathy and back pain by compressing the adjacent neural structures. ESS is an effective minimally invasive technique for treating lumbar synovial cysts (LSCs). This approach involves the use of small endoscopes and tubular retractors to visualize and excise the cysts, with minimal disruption to the surrounding tissues. Studies have demonstrated that endoscopic resection of LSCs can achieve favorable outcomes, with reduced postoperative pain and faster recovery times compared to those for traditional open surgery. For instance, a systematic review and meta-analysis by Garg and Kasliwal [68] found that 86% of patients undergoing minimally invasive excision of LSCs, including endoscopic techniques, had favorable outcomes, according to Macnab’s criteria, with significant improvements in the Oswestry Disability Index (ODI) and Visual Analog Scale (VAS) scores. In addition, Oertel and Burkhardt reported that 81.8% of patients treated with endoscopic tubular-assisted resection of LSCs experienced excellent or good clinical outcomes, with significant reductions in leg and back pain [69]. Endoscopic techniques can allow for complete cyst removal, while still preserving the surrounding structures.

## 6. Failed Back Surgery Syndrome

Failed back surgery syndrome (FBSS) is a general term for persistent postoperative back pain, with or without accompanying radicular pain [70]. It occurs after one or more back surgeries involving the same or adjacent intervertebral spaces, usually involving the lumbar and sacral spine. Patients with this condition complain of chronic back pain that radiates to the lower limbs. 

Traditional treatment for failed back surgery syndrome (FBSS) typically involves open surgery for nerve decompression, potentially resulting in increased instability and back pain, necessitating fusion [71]. A commonly employed technique is PLIF. However, this technique is associated with dural tears and neurologic injury. Conservative treatments include the use of non-steroidal anti-inflammatory drugs (NSAIDs), muscle relaxants, tricyclic antidepressants, and acetaminophen, although opioids and NSAIDs, while effective, can be accompanied by numerous adverse effects on the renal and gastrointestinal system [72].

Alternative treatments include spinal cord stimulation (SCS), which uses pulsed electrical signals near the spinal cord to reduce pain and improve mobility, although it may lead to complications like infection or hematoma. Dorsal root ganglion (DRG) stimulation at L2–L3 offers an effective alternative, while peripheral nerve stimulation (PNS), subcutaneously targeting nerves, presents fewer complications than SCS [72].

Percutaneous endoscopic lumbar discectomy (PELD) is a promising procedure due to its minimally invasive nature, necessitating only a small incision and resulting in quick recovery and short hospital stay. In addition, its efficacy has been shown to be equivalent to that of open surgery. Nevertheless, there can be complications with this procedure, such as dural tear, nerve root injury, recurrence, and others [73]. Furthermore, epiduroscopy is a less known procedure that focuses on breaking down epidural adhesions that form around the affected nerve. This is treated with the mechanical movement of a catheter that helps loosen the adhesions and saline injections around the affected nerve root to help break down the adhesions, with the hope of reducing inflammation around the nerve. Afterward, drugs such as corticosteroids can be delivered to the affected area.

Compared to open surgery, epiduroscopy is a promising procedure to treat FBSS; it is minimally invasive, resulting in a faster recovery time and less risk of complications. It also provides a clear inspection of the epidural space, allowing for the identification of adhesions that may not be seen on conventional imaging tests. Furthermore, it has shown significant pain relief and increased quality of life for patients with FBSS, as emphasized in a 2021 meta-analysis by Geudeke et al. [74], which included 392 patients. A systematic review by Hayek et al. [75] evaluated the effectiveness and safety of spinal endoscopic adhesiolysis in managing chronic low back and lower extremity pain in post-surgical patients with FBSS. The study included one randomized control trial and five observational studies, concluding that it might be an effective treatment modality for chronic refractory low back pain and radiculopathy related to epidural adhesions. An update by Helm et al. [76] also reviewed the effectiveness of spinal endoscopic adhesiolysis in treating post-lumbar surgery syndrome. They found fair evidence supporting the effectiveness of the procedure for persistent low back or leg pain in FBSS patients.

Nevertheless, there are many barriers to the clear-cut implementation of this procedure for patients with FBSS. Major obstacles include the technical demand and the risks involved with this procedure. Navigating through the epidural space may not be an easy task, and the neurosurgeon must be precise to avoid any major complications such as damaging nerve roots or causing dural tears. In addition, the effectiveness is irregular, as some patients experience only temporary relief. Finally, there is a chance of complications such as infection, bleeding, and fibrosis recurrence post-procedure.

## 7. Spinal Tumors

One of the earlier records regarding the use of endoscopic spine surgery (ESS) in tumor surgery was the case series published by Rosenthal et al. in the 1990s, where the authors present the advantages of microsurgical endoscopy over classic thoracotomy in the removal of ventral spinal metastases [77]. The use of this technique resulted in reduced surgical trauma to tissues, increased pain relief due to effective decompression, restoration of spinal stability and alignment, quicker recovery, and a shorter convalescence period. Despite the initial success, the lack of literature comparisons with the traditional open approach motivated the authors not to consider ESS as a valid substitute, but only as a potentially valuable alternative in the treatment of thoracic spine disease.

The primary objectives of surgical management for spine tumors are local disease control and at least one year of survival for any spinal metastases [78]. The most effective course of treatment for the pain and neurological symptoms brought on by spinal instability is surgery. The following conditions are indications for surgery: radiotherapy-resistant malignancies; neurological deficiency before, during, or after the radiation therapy; vertebral collapse, with or without neurological deficit; excruciating pain that is not responsive to conventional therapy (medication, radiation, minimally invasive stabilizing interventions, etc.); and spinal instability [79].

The advancement of endoscopic equipment and techniques has gradually broadened the indications for ESS, prompting interest and exploration within the spine surgery community, particularly in the field of spinal oncology [80]. 

Despite its growing use in the treatment of different spinal pathologies, the application of ESS for tumors remains restricted and difficult, particularly for large, highly vascularized, or intradural lesions. During the procedure, it is crucial to precisely and bilaterally slice the tissue plane that separates the tumor from the surrounding tissue. The dissection method necessitates two-handed cooperation, making tumor dissection a difficult procedure for either uniportal or biportal endoscopic surgery. Furthermore, in highly vascularized tumors, hemostasis under endoscopic vision can be challenging [11].

However, there have been reported cases of successful ESS for the symptomatic decompression of the dural sac or other neural structures in cases of spinal metastasis [81] for which the primary goal of the surgery is restoring neurological function rather than a radical resection of the mass [82].

The use of ESS for intraspinal malignancies displays a potentially bright future, as a number of significant technological developments, such as real-time neuronavigation and high-resolution imaging systems, are making tumor resections more precise, safer, and more efficient, and this will probably broaden its applications in oncological care [10]. Furthermore, the incorporation of robotic-assisted systems has the potential to improve the precision and control of the manipulating instruments, especially in cases involving intricate anatomy [83]. ESS is anticipated to be used in more difficult tumor types and locations as surgical methods and technologies advance [84]. 

To guarantee that the upcoming generation of surgeons is well equipped with these minimally invasive techniques, training schools are progressively including ESS in their curricula for spine surgery [2].

However, difficulties remain, such as the expensive equipment required, making the tools less widely available [85], and the difficult learning curve required to become proficient in these methods [86]. Furthermore, even though the preliminary findings are positive, longer-term data with greater detail are required to validate the effect of ESS on survival rates and tumor recurrence.

Notwithstanding these challenges, ESS appears to have a promising future, as it provides the possibility of extremely accurate, minimally invasive operations that achieve better patient outcomes, reduced risk of adverse events, and decreased time and costs of hospitalization.

## 8. Technical Challenges and Future Outlook

ESS represents a turning point for spinal care and offers minimally invasive procedures that can effectively address a wide range of common spinal pathologies. The numerous advantages that come with ESS are accompanied by a large array of technical challenges and drawbacks that will be faced by surgeons, limiting its widespread adoption [87].

**Figure 3:**
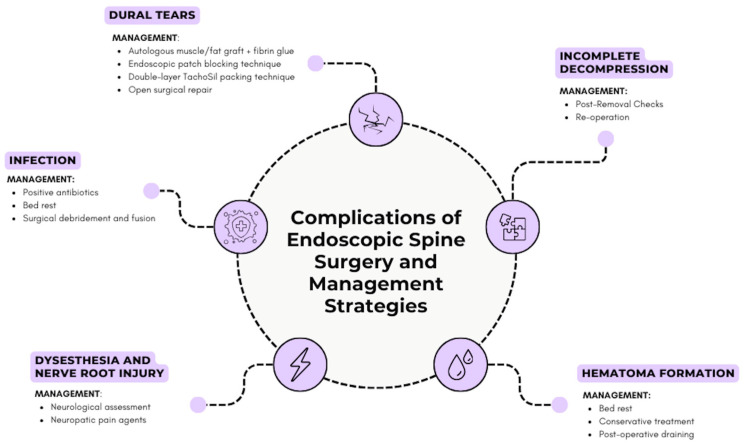
Diagram showing potential complications of ESS and their management strategies. (created by the authors with Canva.com).

Firstly, one of the primary disadvantages of endoscopic spine surgery (ESS) lies in its reliance on specialized equipment, including advanced endoscopes with high-definition optics, next-generation navigation systems, and increasingly, robotic-assisted platforms, which come with substantial costs [88]. The high expense associated with acquiring and maintaining this equipment limits accessibility, especially for smaller or budget-constrained healthcare facilities and in regions with restricted healthcare funding [88]. For many hospitals, these costs represent a significant financial commitment, often limiting ESS’s adoption to specialized centers. ESS offers shorter operation times, reduced complication rates, and decreased in-hospital stays [89]. Despite these benefits, the total costs associated with ESS currently remain higher than those of traditional open surgery [89], and many risks still accompany the endoscopic approach (Figure 3). However, hospital expenses are more significantly influenced by the greater length of stay and higher frequency of readmissions—factors more commonly associated with open surgical procedures [89]. As technological advancements continue and equipment production scales up, driving costs down, ESS may increasingly emerge as the preferred, cost-effective option for spinal surgery [84]. 

Secondly, another inherent limitation of ESS is the lack of stereognosis and the limited depth perception offered by traditional two-dimensional (2D) endoscopic imaging [90]. This reduction in spatial awareness increases the risk of vascular and neural injuries, as surgeons may find it challenging to accurately gauge distances and navigate around critical structures within the spine. The reliance on 2D imaging can be especially problematic in delicate areas with complex anatomy, where precision is paramount to prevent damage to blood vessels and nerves. In addition, despite improvements in optics and imaging, maintaining clear visualization of critical structures remains challenging, especially in cases with highly vascularized or dense tissues [6]. Achieving effective hemostasis, as a consequence of poor vessel visualization during endoscopic procedures, is particularly difficult, often complicating the surgery and potentially extending operative times [91]. To address these challenges, three-dimensional (3D) endoscopic systems have recently been introduced, particularly in procedures for lumbar degenerative disease [92,93]. These 3D systems provide enhanced visual depth, allowing for a more comprehensive and accurate depiction of pathological lesions, improving spatial awareness, and potentially reducing the risk of intraoperative injury [94]. By offering surgeons a clearer view of the anatomy, 3D endoscopy enables more precise navigation and manipulation within confined spaces, and it may improve surgical outcomes in ESS and decrease operative times [95]. 

Finally, endoscopic spine surgery (ESS) demands a notably steep learning curve, primarily due to the specialized skills and technical proficiency required to perform these procedures effectively [86]. Surgeons must master unique visual and spatial skills, including the ability to interpret endoscopic images that provide magnified, but often inverted, views of the spine’s anatomy [86,96]. This setup fundamentally changes the surgeon’s relationship with the operating field, as they must rely on indirect visualization rather than direct, three-dimensional observation, creating significant cognitive demands during initial training [97]. Furthermore, the specialized equipment required for endoscopic procedures varies from the that used for open surgeries. ESS surgeons must achieve great maneuverability with long, thin graspers, rongeurs, and burrs in order to avoid unintended contact with neural structures [86]. Surgeons must learn to work with angled optics, manage fluid irrigation to maintain visualization, and navigate ergonomically challenging setups, all of which add to the technical demands of ESS [86]. Reaching proficiency in ESS can require completing anywhere from 15 to 80 cases, depending on the individual surgeons’ adaptation capacity and the complexity of the cases [96]. To mitigate the steep learning curve of endoscopic spine surgery (ESS), a combination of advanced training methods is essential [97]. Simulation-based programs, virtual reality and augmented reality platforms, and cadaveric labs offer immersive, hands-on experiences that build critical skills, such as hand–eye coordination and precise instrument handling [98,99,100]. Real-time navigation systems and AR may reduce radiation exposure by minimizing the need for repeated fluoroscopy [101], while also improving trajectory planning and safety margins. AR is being increasingly explored and used in the world of neurosurgery [102], and robotic guidance platforms show potential to enhance accuracy, thereby reducing the technical demands on new surgeons and potentially flattening the learning curve [103].

Mentorship and proctorship programs provide real-time feedback, while mini-fellowships and workshops offer intensive, focused training which is essential for both residents and senior physicians to acquire the knowledge behind the complex techniques of ESS [104]. Online modules and emerging certification programs create a structured learning path, ensuring competency and consistency in ESS practices [105]. Together, these multifaceted training approaches equip surgeons to perform ESS safely and effectively, potentially leading to broader adoption and improved patient outcomes.

Overall, ESS represents a pivotal advancement in the field of minimally invasive spinal surgery; nevertheless, its widespread employment is restricted by different technical limitations which can be reduced and eliminated in the near future. While ESS offers reduced surgical trauma, shorter operative and recovery times, and lower complication rates compared to those for traditional open surgery, the approach is hindered by the high cost and complexity of specialized equipment. Furthermore, the steep learning curve required for proficiency associated with highly specialize surgical equipment, indirect visualization, and complex spatial awareness, together with the absence of a specific ESS program in residency school, makes the procure available only for well-trained physicians. However, ongoing innovations in visualization technology, robotic assistance, and surgeon training hold promise for addressing these challenges. As these advancements continue to evolve, ESS may become increasingly accessible and cost-effective, positioning it as a transformative option in the future landscape of spinal care (Table 1). 

## Figures and Tables

**Table 1:** Table summarizing the most common indications, contraindications, applications, and outcomes of ESS.

**Application**	**Indications**	**Contraindications**	**Common Applications**	**Estimated Approximate Complication Rate (According to Meta-Analyses)**	**Expected Outcomes**	**ESS Compared to Open**
Spinal Stenosis	- Chronic pain and limited mobility due to the narrowing of the spaces surrounding spinal neurovascular structures. - Unsuccessful conservative treatment options.	- Pure back pain, with no neurogenic symptoms. - Instability/deformities requiring stabilization. - Complex stenosis.	- Endoscopic decompression. - Endoscopic fusion.	≈8.1%	- Less perioperative blood loss. - Reduced postoperative pain. - Reduced hospital stay.	***ESS Advantages:*** - *Minimally invasive.* - *Shortened recovery time.* - *Reduced post-op risks.* - *Reduced hospital stays.* - *Potential decrease in anesthesia-related risks.*
Thoracic Disc Herniation	- Symptomatic TDG with spinal cord compression. - Persistent pain, despite conservative treatments. - Calcified discs requiring minimally invasive access.	- Severe thoracic canal stenosis. - Complex anatomical structures limiting access. - High risk of vascular or pulmonary injury.	- Transforaminal endoscopic thoracic discectomy. - Endoscopic interlaminar approach. - Transthoracic retropleural approach.	Dural tear: ≈1.3% Dysesthesia: ≈4.7% Recurrent herniation: ≈2.9% Myelopathy: ≈2.1% Epidural Hematoma: ≈1.1%	- Effective decompression, with minimal tissue disruption. - Reduced complication rates. - Decreased hospitalization duration and faster recovery.	***Open Advantages:*** - *Greater visibility.* - *Established technique.* - *Direct access.* - *Easier handling of complications.*
Spondylolisthesis, Degenerative Conditions, and Synovial Cysts	- Chronic back pain or nerve compression due to vertebral slippage. - Failed conservative treatment options.	- Severe osteoporosis or spinal instability. - Complex anatomical variations.	- Endoscopic decompression. - Endoscopic fusion. - Endoscopic tubular-assisted resection (for cysts).	*Varies greatly according to the condition;* Durotomies: ≈2.23% (8% for cysts) Inadequate decompression: ≈1.29% (cyst recurrence: 4%, cyst re-operation: 5%) Epidural Hematomas: ≈3.79% Transient nerve root injuries: <1%	- Reduced postoperative pain. - Faster recovery time. - Decreased hospitalization duration.	
Failed Back Surgery Syndrome	- Minor or moderate disc herniation or nerve compression. - Persistent pain. - No response to conservative management.	- Severe spinal degeneration. - Complex herniations. - High risk of infection or bleeding.	- Percutaneous endoscopic lumbar discectomy (PELD). - Epiduroscopy. - Minimally invasive decompression.	N/A	- Short recovery time. - Fast pain relief. - Less dependence on pain medications.	
Spinal Tumor Resection	- Radiotherapy-resistant malignancies. - Severe pain unresponsive to conservative treatment. - Spinal instability.	- Large or highly vascularized tumors. - Intradural lesions. - Severe vertebral collapse.	- Metastatic tumor resection. - Selected cases of limited primary tumors.	≈6.56%	- Pain relief and decompression. - Quicker recovery and reduced hospital stay. - Improved mobility and quality of life.	

## Data Availability

This study is a narrative review and does not involve the generation or analysis of new data. All data discussed are derived from publicly available sources cited within the manuscript. As such, there are no additional datasets to share.

## Abbreviations

The following abbreviations are used in this manuscript:

ALIF	anterior lumbar interbody fusion
BMPs	bone morphogenetic proteins
DRG	dorsal root ganglion
ESS	endoscopic spine surgery
FBSS	failed back surgery syndrome
FESS	full-endoscopic spine surgery
LDH	lumbar disc herniation
LSS	lumbar spinal stenosis
NSAIDs	non-steroidal anti-inflammatory drugs
PELD	percutaneous endoscopic lumbar discectomy
PLIF	posterior lumbar interbody fusion
SCS	spinal cord stimulation
TDH	thoracic disc herniation
TETD	transforaminal endoscopic thoracic discectomy
TLIF	transforaminal lumbar interbody fusion
UBE	unilateral biportal endoscopy
